# Obstetric history after mechanical cardiac valve replacement

**DOI:** 10.1186/1749-8090-10-S1-A239

**Published:** 2015-12-16

**Authors:** Vivek M Kanhere, Anjali Kanhere, Vinod Narkhede

**Affiliations:** 1 Department of Cardiothoracic Surgery, Chirayu Medical College & Hospital, Bhopal, Madhya Pradesh, 462030 India; 2 Department of Obstetrics & Gynecology, Peoples Medical College & Hospital, Bhopal, Madhya Pradesh, 462038 India; 3 Department of Community Medicine, Chirayu Medical College & Hospital, Bhopal, Madhya Pradesh, 462030 India

## Background/Introduction

In patients who have undergone mechanical prosthetic heart valve replacement, pregnancy is associated with the risks of warfarin embryopathy. A significant proportion of our patient undergoing mechanical mitral cardiac valve replacement were females of child bearing age.

## Aims/Objectives

To study the obstetric history in patients after mechanical cardiac valve replacement.

## Method

Retrospective analysis of 35 subjects who conceived after cardiac valve replacement with mechanical valves and presented with 45 pregnancies was carried out. Ten patients had multiple pregnancies (2 each). 10 patients had documentary evidence of termination of pregnancy elsewhere outside the institute. Thus we document 35 pregnancies in 25 cases.

## Results

45.7% had successful pregnancy outcome. 40% cases had missed abortion. Five patients chose to have Medical Termination of Pregnancy (MTP) with tubal ligation. The majority of subjects (76.4%) delivered within 5 years of valve replacement. Despite counselling about embryopathy all except one patient continued to take oral anticoagulants. There was no maternal mortality. Morbidity in the form of post-partum hemorrhage (8.5%), bleeding complications (5.7%) was observed. The incidence of preterm delivery was 14.3% and small for gestational age babies were 5.7%. There were no still births; one baby had malformations but was not consistent with features of embryopathy.

**Table 1:** Pregnancy outcome in study subjects

Pregnancy Outcome	No. of Subjects	Percentage (%)
MTP	05	14.3
Abortion	14	40
Full Term Normal Delivery	11	31.4
Cesarean Section	05	14.3

## Discussion/Conclusion

Significant proportion of patients having valve replacement in our centre are females. Post-surgery their obstetric history is complicated by poor awareness about oral anticoagulation and its effect on pregnancy this results in delay in seeking appropriate obstetric care. In pregnancy there is an increased fetal wastage in patients taking oral anticoagulants though women with artificial valves can tolerate the hemodynamic load of pregnancy well. A successful pregnancy outcome in women with surgically corrected valve pathology involves preconception evaluation, counseling, close supervision during her pregnancy and labor.

